# Association between bacterial vaginosis and 25-Hydroxy vitamin D: a case-control study

**DOI:** 10.1186/s12879-023-08120-3

**Published:** 2023-04-06

**Authors:** Seyede Faezeh Mojtahedi, Alireza Mohammadzadeh, Fatemeh Mohammadzadeh, Jelveh Jalili Shahri, Narjes Bahri

**Affiliations:** 1 Student Research Committee, Faculty of Medicine, Gonabad University of Medical Sciences, Gonabad, Iran; 2 Department of Microbiology, Faculty of Medicine, Infectious Diseases Research Center, Gonabad University of Medical Sciences, Gonabad, Iran; 3 School of health, Social Development and Health Promotion Research Center, Gonabad University of Medical Sciences, Gonabad, Iran; 4 Department of Obstetrics and Gynecology, Faculty of Medicine, Gonabad University of Medical Sciences, Gonabad, Iran; 5 Department of Midwifery, Faculty of Medicine, Social Determinants of Health Research Center, Gonabad University of Medical Sciences, Gonabad, Iran

**Keywords:** Bacterial vaginosis, Vitamin D, Vaginal microbiota, Women health, Case-control study

## Abstract

**Introduction:**

Bacterial vaginosis (BV) is the most common vaginal dysbiosis among women of reproductive age. Micronutrient deficiencies, including vitamin D deficiency, can increase the risk of BV. The findings of previous studies regarding the relationship between vitamin D deficiency and BV were conflicting. Therefore, this study aimed to evaluate the association between BV and serum level of 25-hydroxy vitamin D.

**Materials and methods:**

This case-control study was conducted in Gonabad County in 2021. One hundred and twenty-five confirmed BV cases and 125 controls who were matched based on age and intercourse frequency (maximum difference of two days per week) enrolled in the study. Data collection was performed using a demographic and reproductive data questionnaire and a checklist for recording Whiff test results, serum 25-hydroxy vitamin D level, litmus paper observation, and microscopic findings (clue cells). Serum level of vitamin D was evaluated based on enzyme-linked immunoassay method (Monobind kit) from 0.5 ml venous blood drawn from each participant. The conditional logistic regression model was used to analyze data.

**Results:**

The BV cases had significantly lower 25-hydroxy vitamin D serum levels than controls. The odds of BV increased with vitamin D deficiency (Adjusted odds ratio (AOR): 4.34, 95% confidence interval (CI): 1.39–13.4, p = 0.011, FDR q-value = 0.051), vitamin D insufficiency (AOR: 3.65, 95% CI: 1.23–10.85; p = 0.020; FDR q-value = 0.053), cigarette/hookah smoking (AOR: 3.65, 95% CI: 1.23, 10.85; p = 0.020; FDR q-value = 0.053) and lower age at first intercourse (AOR: 1.16, 95% CI: 1.05, 1.28; p = 0.004; FDR q-value = 0.048). The odds of BV was 0.80 and 0.78 times lower in participants who had coitus interruptus (AOR: 0.20, 95% CI: 0.06, 0.63; p = 0.006; FDR q-value = 0.048) and condom use (AOR: 0.28, 95% CI: 0.10, 0.79; p = 0.016; FDR q-value = 0.051), respectively, compared to participants who did not use contraceptives.

**Conclusion:**

These findings suggested that lower serum vitamin D levels were associated with an increased risk of developing BV. However, further studies are needed to confirm the results of the present study.

## Introduction

Normal vaginal *Lactobacillus* microbiota is a defense mechanism against vaginal dysbiosis by vaginal pathogens [1, 2]. Bacterial vaginosis (BV) is defined as change in normal vaginal microbiota that can reduce the level of hydrogen peroxide producing lactobacilli and overgrowth of anaerobic bacteria and thus increase the risk of sexually transmitted infections. BV is the most common cause of vaginitis among women of reproductive age. The prevalence of BV in the United States and Europe were reported to be 29% and 4–14%, respectively [3, 4]. In Iran, the prevalence of BV among women at reproductive age is reported to range between 16.2% and 37.7% [5, 6]. However, in a meta-analysis study the prevalence of BV was reported to be 28% in Iran [7].

The complications of BV may include increased risk for infection with human immunodeficiency virus (HIV), herpes simplex virus (HSV) type 1 and 2; *Neisseria gonorrhoeae*, and *Chlamydia trachomatis* as well as increased risk of stress, depression, and social withdrawal. On the other hand, BV is associated with increased risk of premature rupture of membranes, chorioamnionitis, and post-natal endometritis in pregnant women [8, 9].

Although a number of predisposing factors, including age, hormonal changes, race, socio-economic status, frequent sexual intercourse, multiple sexual partners, vaginal douching, cigarette, mental stress, and social factors, have been suggested for bacterial vaginosis, the etiology of BV has not yet been clearly identified [10, 11]. Some studies have shown that factors including micronutrient deficiency, including vitamin D deficiency can impair immune function and increase the risk of BV [12, 13]. The findings of studies on pregnant and non-pregnant women in this regard were conflicting. While some studies on pregnant and non-pregnant women reported that serum vitamin D was not associated with increased risk of BV [14, 15], some studies reported a positive relationship between serum level of vitamin D and BV [16, 17].In another study, vitamin D supplementation was found effective in eliminating BV among women of reproductive age [18]. Considering the controversies in the findings of previous studies, there is a need for further evidence in this regard. Therefore, the current study was conducted to evaluate the relationship between serum 25 hydroxy vitamin D level and BV.

## Methods

### Study design

This case-control study was conducted in North-East Iran in 2021. The study report was prepared based on the Strengthening the Reporting of Observational Studies in Epidemiology (STROBE) Statement checklist for case-control studies (2010) [19].

### Setting and participants

A total of 250 married women (125 in the BV group and 125 in the control groups) who were referred to the Bohlol Hospital, private obstetrics and gynecology clinics, and Integrative Health Centers in Gonabad city participated in the current study. The BV group included married women with complaints of vaginal discharge who were suspected of BV. The control group was chosen from women referring to the centers for routine obstetrics and gynecology evaluation, including preconception counseling, contraception, breast or cervical cancer screening, and midlife care. The BV and control groups were matched based on age (maximum difference of 3 years) and frequency of intercourse (maximum difference of 2 days per week) with a matching ratio of 1:1.

Inclusion criteria for the case group were as follows: [1] Not being pregnant, [2] Not suffering from underlying diseases, including diabetes and immunodeficiency, [3] Not taking immunosuppressive drugs, [4] Willingness to participate in the study, and [5] Being suspected to have BV due to vaginal discharge and subsequent confirmation of BV based on laboratory tests.

Inclusion criteria for the control group were similar to the BV group except for complaining of vaginal discharge and BV suspicion. The exclusion criterion was refusing to participate in the study.

### Measurements

The data were collected using a questionnaire including several items about demographic and reproductive characteristics and predisposing factors for BV. Demographic data included participant’s age, spousal age, educational level, spousal educational level, income level, occupation, cigarette/hookah smoking, other questionnaire items included vitamin D supplement consumption, vaginal douching, pregnancy history, delivery history, abortion history, history of premature delivery, history of taking medication for vaginitis, history of cryotherapy for vaginitis, history for cauterization for vaginitis, dairy consumption, menstruation status, family planning method, age at first sexual intercourse, duration of sunlight exposure, frequency of sexual intercourse per week, and serum vitamin D level. Body Mass Index (BMI) was calculated as weight/height2 (kg/m2) and divided into four categories, underweight defined as BMI < 18.5 kg/m2, normal-weight defined as BMI 18.5–24.9 kg/m2, overweight defined as BMI 25.0-29.9 kg/m2, and obese defined as BMI ≥ 30.0 kg/m2, based on the World Health Organization (WHO) criteria [20]. A checklist was also filled for each participant and data regarding the findings of the Whiff test, serum level of 25 hydroxy vitamin D, litmus paper evaluation, and the findings of microscopic evaluation (reporting clue cells) were collected.

### Diagnosis of BV

Vaginal samples were obtained from all participants to evaluate BV. Inspection of the physical appearance of the vagina and vaginal discharge was performed using a sterile speculum. The physical appearance characteristics of vaginal discharge, including shape, consistency, and color, were recorded in the study checklist. Then two smears were obtained from the lateral vaginal wall using two sterile cotton swabs. One swab was used to prepare the smear and was sent to the laboratory, while the second swab was used to evaluate vaginal pH by applying the swab on litmus paper. The litmus paper would have a pink discoloration if BV presents. Whiff test was performed by applying a drop of potassium hydroxide to the vaginal smear slides, and the presence of amine odor was evaluated. Vaginal smears were evaluated for BV in the laboratory. Slides were stained and inspected using a light microscope at 100 X magnification to define the percentage of clue cells. Diagnosis of BV was made based on the presence of at least three out of four Amsel criteria, including homogenous vaginal discharge, vaginal pH greater than 4.5, positive Whiff test, and more than 20% clue cells in the microscopic evaluation of vaginal sample [21].

### Evaluation of serum 25 hydroxy vitamin D

In order to evaluate serum 25 hydroxy vitamin D level, 0.5 ml venous blood was drawn from all participants. Serum 25 hydroxy vitamin D level was determined by 25(OH) vitamin D kit (Monobind Inc. USA) using ELISA method (Automated ELISA system, Dynex DS2, USA). Briefly, a standard curve is drawn by using the average optical absorption of the standards. Then, the vitamin D concentration of the sample is identified considering the average optical absorption of the sample and the standard curve. Two control serum with specific concentrations were used. According to the kit guideline, the criteria used to define vitamin D deficiency were as follows: serum vitamin D < 10 ng/ml was defined as vitamin D deficiency, serum vitamin D between 10 and 30 ng/ml was defined as vitamin D insufficiency, serum vitamin D between 31 and 100 ng/ml was defined as vitamin D sufficiency, and serum vitamin D > 100 ng/ml was defined as vitamin D toxicity.

### Sample size

The study sample size was calculated using the sample size equation for case-control studies [22] based on the reported prevalence of vitamin D deficiency (p = 0.6) from a previous study [23] and considering Type 1 error of 5%, Type 2 error of 20%, and odds ratio (OR) of 2.5. The sample size was increased by 25% based on the confounders. The final sample size was 125 participants in each group.

### Statistical analysis

Qualitative variables were described using frequency and percentage. The normality of the quantitative variables was assessed using Kolmogorov–Smirnov test and skewness and kurtosis values. Normal quantitative variables were described using mean and standard deviation (SD) and non-normal quantitative variables were described using median and 1st and 3rd quartiles. The association between serum 25 hydroxy vitamin D level and BV status was evaluated using a conditional logistic regression model, preferred for matched case-control studies analysis, with adjustment for potential confounding variables. Variables with p-value higher than the cut-off value of 0.15 in simple conditional logistic regression were considered as confounders. False discovery rate (FDR) correction was performed by the Benjamin-Hochberg procedure to account for multiple comparisons using Seed-based d Mapping software (SDM, version 6.21, https://www.sdmproject.com). The statistical significance level was set as p-value < 0.05. For multiple comparison correction, the statistical level was set to FDR q-value < 0.10. The results were reported as odds ratios (ORs) and 95% confidence intervals (CIs) for OR. All statistical analyses were performed using the statistical package for social sciences (SPSS) software version 16 (SPSS Inc., Chicago, Ill., USA) and Stata software, version 12 (Stata Corp, College Station, Texas USA).

### Ethical considerations

The current study was approved by the Ethical Committee of the Gonabad University of Medical Sciences (IR.GMU.REC.1398.150). All participants were informed about the purpose of the research and signed a written informed consent prior to participating in the study. The identity of the participants was kept confidential. All examinations and laboratory tests were free of charge. As an incentive, a free gynecologist visit was arranged for participants with documented BV. Furthermore, participants with vitamin D deficiency were treated.

## Results

### Characteristics of the study population

**Fig. 1:**
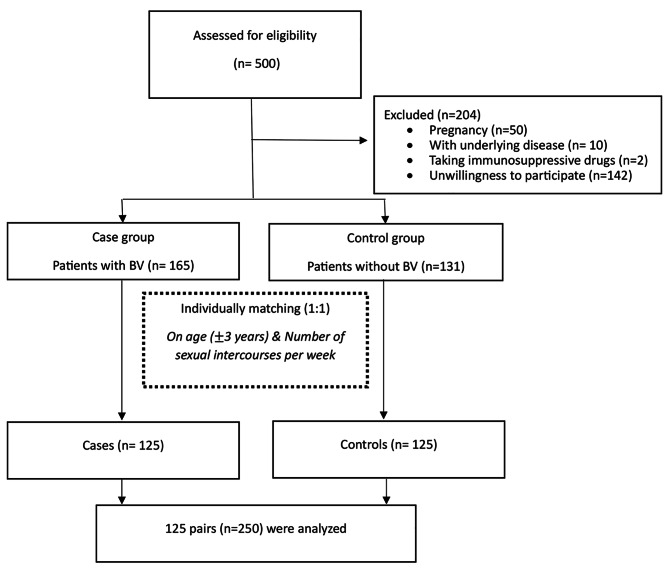
Flow diagram of the study

A total of 135 and 161 women consented to participate in the BV group and control groups, respectively. After matching the BV and control groups based on age and frequency of weekly sexual intercourse, 125 participants were included in each study group. Figure 1 presents the diagram of the study.

Table 1 presents the demographic and reproductive characteristics of the study participants. The mean age of the participants was 35.4 (SD = 9.9) years old, ranging from 17.0 to 63.0 years. The mean BMI was 25.6 (SD = 4.1) kg/m^2^. Mean age at first sexual intercourse was 35.4 (SD = 9.9) years old. Approximately half of the participants (48.4%) had a university degree. The income level of 81.4% of the participants were sufficient to support the family. Most of the women (62.4%) were housewives, while 11.6% were cigarettes/hookah smokers, 24.8% regularly used vitamin D supplements, 77.6% consumed dairy products daily. The mean frequency of intercourse of the participants was 2.0 (SD = 1.1) times per week. Regarding family planning methods, 31.2% of the participants had no contraception and 30.4% used condoms.

**Table 1:** Demographic and reproductive characteristics of the participants

Variable	BV group	Control group
**Age (years), Mean (SD)**	35.4 (9.9)	35.3 (9.8)
**Spousal age (years), Mean (SD)**	40.2 (10.7)	39.8 (10.7)
**Educational level, n (%)**		
Below high school	31 (24.8)	37 (29.6)
High school graduate	29 (23.2)	31 (24.8)
Associate’s or bachelor’s degree	54 (43.2)	52 (41.6)
Master’s or higher degree	11 (8.8)	4 (4.0)
**Spousal educational level, n(%)**		
Below high school	33 (26.4)	28 (22.4)
High school graduate	38 (30.4)	38 (30.4)
Associate’s or bachelor’s degree	46 (36.8)	50 (40.0)
Master’s or higher degree	8 (6.4)	9 (7.2)
**Income level, n (%)**		
Not enough to support the family adequately	23 (18.4)	23 (18.4)
Enough to support the family adequately	102 (81.6)	102 (81.6)
**Occupation, n (%)**		
Housewife	75 (60.0)	81 (64.8)
Employee	38 (30.4)	36 (28.8)
Home-based businesses	12 (9.6)	8 (6.4)
**BMI, n (%)**		
Underweight	2 (1.6)	5 (4.0)
Normal weight	55 (44.0)	53 (42.4)
Overweight	52 (41.6)	42 (33.6)
Obese	16 (12.8)	25 (20.0)
**Cigarette/Hookah smoking, n (%)**		
Yes	21 (16.8)	8 (6.4)
No	104 (83.2)	117 (93.6)
**Vitamin D supplement use, n (%)**		
Regular	27 (21.6)	35 (28.0)
Irregular	36 928.8)	37 (29.6)
No consumption	62 (49.6)	53 (42.4)
**Sunlight exposure (minutes/day), Median (1st ,3rd quartiles)**	60.0 (30.0, 90.0)	60.0 (30.0, 120.0)
**Dairy consumption, n (%)**		
Never or rarely	29 (23.2)	27 (21.6)
Less than 2 servings/day	64 (1.2)	63 (50.4)
Two or more servings/day	32 (25.6)	35 (28.0)
**Age at first sexual intercourse (years) ), Mean (SD)**	19.0 (3.7)	20.7(4.8)
**Frequency of intercourse (per week), Mean (SD)**	2.0 (1.1)	2.0 (1.1)
**History for cryotherapy for vaginitis, n (%)**		
Yes	13 (10.4)	15 (12.0)
no	112 (89.6)	110 (88.0)
**History for cauterization for vaginitis, n (%)**		
Yes	7 (5.6)	2 (1.6)
no	118 (94.4)	123 (98.4)
**History of medication taking for vaginitis, n (%)**		
Yes	104 (83.2)	93 (74.4)
No	21 (16.8)	32 (25.6)
**Pregnancy history, n (%)**		
Yes	98 (78.4)	104 (83.2)
no	27 (21.6)	21 (16.8)
**Delivery history, n (%)**		
Yes	99 (79.2)	104 (83.2)
no	26 (20.8)	21 (16.8)
**Abortion history, n (%)**		
Yes	30 (24.0)	27 (21.6)
No	95 (76.0)	98 (78.4)
**Premature delivery, n (%)**		
Yes	13 (10.4)	21 (16.8)
No	112 (89.6)	104 (83.2)
**Vaginal douching, n (%)**		
Yes	40 (32.0)	54 (43.2)
No	85 (68.0)	71 (56.8)
**Menstruation status**		
Regular	84 (67.2)	89 (71.2)
Irregular	27 (21.6)	26 (20.8)
Menopause	14 (11.2)	10 (8.0)
**Family planning methods, n (%)**		
No contraception	47 (37.6)	31 (24.8)
Coitus Interruptus	15 (12.0)	30 (24.0)
Condom	34 (27.2)	42 (33.6)
Hormonal	10 (8.0)	4 (3.2)
IUD	12 (9.6)	11 (8.8)
Tubal ligation	4 (3.2)	6 (4.8)
Vasectomy	3 (2.4)	2 (1.6)
**Serum vitamin D, n (%)**		
Deficient	67 (53.6)	66 (52.8)
Insufficient	43 (34.4)	35 (28.0)
Sufficient	15 (12.0)	24 (19.2)

Notes: BV, Bacterial vaginosis; SD, Standard deviation

### Serum levels of 25-Hydroxy vitamin D

The mean level of the serum 25 hydroxy vitamin D was 19.7 (SD = 9.6) ng/ml. More than half of the participants (53.2%) had vitamin D deficiency and 31.2% had insufficient serum 25 hydroxy vitamin D levels. The frequency of 25-hydroxy vitamin D sufficient, insufficient and deficient participants is presented in Table 1. The frequency of participants with sufficient 25-hydroxy vitamin D was lower in the BV group (12%) compared to the control group (19.2%).

### The association between bacterial vaginosis and serum levelsof 25-Hydroxy vitamin D

#### Simple conditional logistic regression

Based on the simple conditional logistic regression model, cigarettes or hookah smokers have 4.25 times the odds of having BV than non-smokers (OR = 4.25, p = 0.009). Furthermore, for every one-year increase in the age at first sexual intercourse, the odds of having BV decrease by a factor of 0.89 (OR = 0.89, p = 0.003). Individuals who used the coitus interruptus method (OR = 0.29, p = 0.004) or used condoms (OR = 0.45, p = 0.036) are less likely to have BV than those who did not use contraception.

Cigarette/hookah smoking, vaginal douching, premature delivery, history of taking medication for vaginitis, history of cryotherapy for vaginitis, menstruation status, family planning methods, and age at first sexual intercourse had p-values < 0.15 and were entered into the multiple conditional regression model (Table 2).

**Table 2:** Factors associated with bacterial vaginosis based on the conditional logistic regression analyses

Variable	Simple conditional logistic regression				Multiple conditional logistic regression				
	OR unadjusted	95% CI		P-value	OR adjusted	95% CI		P-value	FDR-adjusted P-value
		LL	UL			LL	UL		
**Age**	1.10	0.90	1.35	0.328	---	---	---	---	---
**Spousal age**	1.03	0.96	1.10	0.366	---	---	---	---	---
**Educational level**									
High school or below	1.38	0.78	2.42	0.260	---	---	---	---	---
University degree	Reference				Reference				
**Spousal educational level**									
High school or below	0.84	0.50	1.40	0.516	---	---	---	---	---
University degree	Reference				Reference				
**Income level**									
Not enough to support the family adequately	1.00	0.52	1.88	1.00	---	---	---	---	---
Enough to support the family adequately	Reference				Reference				
**Occupation**									
Housewife	1.31	0.72	2.38	0.367	---	---	---	---	---
Employed	Reference				Reference				
**Cigarette/Hookah Smoking**									
Yes	4.25	1.43	12.63	0.009	6.15	1.44	26.13	**0.014**	**0.051**
No	Reference				Reference				
**Vitamin D supplement use**									
Regular	1.30	0.63	2.68	0.464	---	---	---	---	---
Irregular	1.58	0.81	3.06	0.173	---	---	---	---	---
No consumption	Reference				Reference				
**Vaginal douching**									
Yes	1.60	0.95	2.70	0.073	1.97	0.99	3.94	0.052	0.119
No	Reference				Reference				
**Pregnancy history**									
Yes	0.64	0.30	1.38	0.261	---	---	---	---	---
No	Reference				Reference				
**Delivery history**									
Yes	0.68	0.31	1.48	0.339	---	---	---	---	---
No	Reference				Reference				
**Abortion history**									
Yes	1.13	0.64	1.96	0.675	---	---	---	---	---
No	Reference				Reference				
**Premature delivery**									
Yes	0.55	0.25	1.20	0.136	0.39	0.14	1.08	0.072	0.140
No	Reference				Reference				
**History of medication taking for vaginitis**									
Yes	2.0	0.24	1.03	0.061	0.48	0.20	1.14	0.100	0.160
No	Reference				Reference				
**History of cryotherapy for vaginitis**									
Yes	0.86	0.41	1.85	0.706	0.21	0.02	1.91	0.168	0.244
No	Reference				Reference				
**History for cauterization for vaginitis**									
Yes	3.57	0.72	16.8	0.118	---	---	---	---	
No	Reference				Reference				
**Dairy consumption**									
Never or rarely	0.95	0.52	1.75	0.892	---	---	---	---	---
Less than 2 servings/day	0.84	0.41	1.72	0.646	---	---	---	---	---
Two or more servings/day	Reference				Reference				
**Menstruation status**									
Regular	0.18	0.02	1.62	0.128	0.34	0.01	8.46	0.513	0.586
Irregular	0.21	0.02	1.88	0.166	0.24	0.01	5.62	0.391	0.481
Menopause	Reference				Reference				
**Family planning methods**									
Coitus Interruptus	0.29	0.12	0.67	0.004	0.20	0.06	0.63	0.006	**0.048**
Condom	0.45	0.21	0.94	0.036	0.28	0.10	0.79	0.016	**0.051**
Hormonal	1.25	0.34	4.51	0.34	1.11	0.24	5.17	0.889	0.889
IUD	0.58	0.19	1.78	0.348	0.48	0.13	1.81	0.285	0.380
Tubal ligation	0.40	0.10	1.55	0.187	0.15	0.01	1.23	0.079	0.140
Vasectomy	0.94	0.14	6.12	0.951	0.54	0.03	7.72	0.653	0.696
No contraception	Reference				Reference				
**Age at first sexual intercourse**	0.89	0.83	0.96	0.003	0.86	0.78	0.95	**0.004**	**0.048**
**Duration of sunlight exposure**	0.99	0.99	1.00	0.371	---	---	---	---	---
**BMI**	0.98	0.92	1.05	0.741	---	---	---	---	---
**Frequency of sexual intercourse per week**	0.66	0.11	3.98	0.657	---	---	---	---	---
**Serum vitamin D level**									
Deficient	1.77	0.79	3.95	0.161	4.34	1.39	13.54	**0.011**	**0.051**
Insufficient	2.07	0.90	4.73	0.084	3.65	1.23	10.85	**0.020**	**0.053**
Sufficient	Reference				Reference				

Notes: OR, Odds ratio; CI, Confidence interval; FDR, False discovery rate; IUD: intrauterine device, BMI: body mass index, UTI: urinary tract infection

#### Multiple conditional logistic regression

After adjusting for potential confounding variables in the multiple conditional logistic regression model, a significant relationship was found between serum 25-hydroxy vitamin D level and BV. This finding showed that individuals with vitamin D deficiency and vitamin D insufficiency are respectively 4.3 (95% CI: 1.39–13.4, p = 0.011, FDR q-value = 0.051) and 3.6 (95% CI: 1.23–10.85; p = 0.020; FDR q-value = 0.053) times more likely to have BV than participants with normal vitamin D levels. Furthermore, the logistic regression model showed that the odds of having BV for cigarette or hookah smokers are 6.15 times as high as the odds for non-smokers (95% CI: 1.23, 10.85; p = 0.020; FDR q-value = 0.053). Participants who had first sexual intercourse at an older age had lower odds for BV. For each one-year increase in the age at first sexual intercourse, the odds of BV reduced by a factor of 0.86 (95% CI: 0.78, 0.95; p = 0.004; FDR q-value = 0.048). In individuals who used the coitus interruptus method (OR = 0.29, 95% CI: 0.06, 0.63; p = 0.006; FDR q-value = 0.048) or used condoms (OR = 0.45, 95% CI: 0.10, 0.79; p = 0.016; FDR q-value = 0.051) are less likely to have BV than those who did not use contraception.

## Discussion

The finding of the current study indicated a significant relationship between serum 25-hydroxy vitamin D levels and BV. The odds of BV for participants with vitamin D deficiency or insufficiency were higher compared to participants with normal vitamin D levels.

Previous studies have shown that sufficient vitamin D can protect women against BV through the production of Cathelicidins, antimicrobial peptides that exist in the lysosomes of macrophages and neutrophiles. Therefore, it is hypothesized that vitamin D may activate pathogen removal mechanisms [8].

A study reported 65% increase in the prevalence of BV among African-American women who had insufficient vitamin D levels compared to those with sufficient vitamin D levels [8]. Another case-control study reported that the serum level of 25-hydroxy vitamin D was significantly lower in participants with BV compared to healthy participants [24]. In a study on 208 Iranian women with BV, vitamin D supplementation at a dose of 2000 international units (IU) per day significantly improved BV treatment response compared to placebo (OR = 10.1, 95% CI for OR: 21, 28.8) [18]. Similarly, Hensel et al., reported a significant relationship between serum 25-hydroxy vitamin D level and BV (OR = 2.87) [15]. In contrast to the current study findings, two previous studies found no relationship between serum 25-hydroxy vitamin D and BV among healthy urban female citizens [14, 25]. The difference in the findings of the studies might be because of the difference in sample size, study design, data analysis methods, geographical distribution, socio-economic, and behaviors between the study populations.

Furthermore, the current study found a significant relationship between cigarette and hookah smoking and BV, indicating that BV is more likely to occur in cigarette or hookah smokers. These findings were in line with the findings of previous studies [14, 26]. Various compounds in cigarettes or hookah smokers reduce the number of hydrogen peroxide producing lactobacilli and change vaginal microbiota. Furthermore, Langerhans cell destruction in the cervical epithelium can result in local immune suppression and BV [14, 27].

This study also found that participants that had sexual intercourse at older age had a significantly lower odds of BV compared to those who had their first sexual intercourse at younger age. This finding was similar to the reported findings of a previous study that reported younger age at first sexual intercourse was a risk factors for BV [27].

In the current study, participants who used natural contraception and condoms had lower odds of BV compared to participants who did not use contraception. In contrast to the findings of the current study, Ranjit et al. reported that the odds of BV was higher in participants who used an intrauterine contraceptive device (IUD) compared to those who did not used an IUD [26]. The discrepancy in results may be due to Ranjit et al., combining copper IUD and hormone-releasing IUD use. Furthermore, Ranjit et al. did not include the duration of IUD use and collected data based on self-report regarding the use of single or multiple contraceptive methods. Participants who used hormone releasing IUDs had the lowest infection rate (9.1%) in the study by Ranjit et al. Similarly, in the study on female sex workers by Jesper et al. in Kenia, sexual intercourse without condom 14–72 h prior to testing increased the odds of BV [28]. Another study also reported that condom use reduced the odds of BV [29]. These findings indicate the importance of condom use. Thus, condoms may prevent alterations to the vaginal microflora. Seminal fluid is alkaline and can buffer the pH of the vagina and cause imbalance in vaginal micro flora. Therefore, less exposure to seminal fluid in individuals who use condoms or coitus interruptus family planning method might explain our findings.

One of the limitations of the current study was including only volunteer participants who referred to the health center for routine care as control group. Therefore, regardless of the acceptable coverage of primary health care in Iran, the findings of the current study may not be generalizable to the total population. Furthermore, the current study evaluated risk factors based on self-report that might result in recall bias. The commercial kit used for the measurement of vitamin D is also prone to quantification error. Considering the simultaneous evaluation of serum 25-hydroxy vitamin D level and BV, the current study might be prone to reverse causation. As limited studies were conducted regarding BV in non-pregnant women in Iran, it is recommended to conduct more studies to reach a definite conclusion.

## Conclusion

The overall findings of the current study showed a significant relationship between serum level of 25-hydroxy vitamin D and BV, indicating that BV in participants with vitamin D deficiency and insufficiency is more likely to occur compared to participants with normal serum vitamin D. However, further studies are needed to confirm the results of the present study.

## Data Availability

The datasets generated and analysed during the current study are not publicly available due to ethical concerns but are available from the corresponding author on reasonable request.

## References

[1] Chee WJY, Chew SY, Than LTL. Vaginal microbiota and the potential of Lactobacillus derivatives in maintaining vaginal health. Microb Cell Fact 2020 Nov 7;19(1):203. doi: 10.1186/s12934-020-01464-4. 10.1186/s12934-020-01464-4 PMC7648308 33160356

[2] Redelinghuys MJ, Geldenhuys J, Jung H, Kock MM (2020). Bacterial vaginosis: current diagnostic avenues and future opportunities. Front Cell Infect Microbiol.

[3] Goodarzi F, Hosseini M (2014). Evaluation of demografic and clinical characters of patients whit bacterial vaginosis in Ahwaz Amir Almoomenin hospital. J Fasa Univ Med Sci.

[4] Muñoz-Barreno A, Cabezas-Mera F, Tejera E, Machado A. Comparative effectiveness of treatments for bacterial vaginosis: a Network Meta-Analysis. Volume 10. Antibiotics; 2021. p. 978. 10.3390/antibiotics10080978 PMC8388924 34439028

[5] Amini B, Baghchesaraie H, Torabi Z. Prevalence of bacterial vaginosis and impact of genital hygiene practices in non-pregnant women in zanjan, iran. Oman Med J 2009 Oct;24(4):288–93doi: 10.5001/omj.2009.58. 10.5001/omj.2009.58 PMC3243866 22216382

[6] Ashraf-Ganjui T, Shahabi M (2003). Epidemiology and risk factors of acterial vaginosis in women visiting the gynecologic clinic of Bahonar Hospital of Kerman University of Medical Sciences. J Kerman Univ Med Sci.

[7] Sabour S, Arzanlou M, Vaez H, Rahimi G, Sahebkar A, Khademi F (2018). Prevalence of bacterial vaginosis in pregnant and non-pregnant iranian women: a systematic review and meta-analysis. Arch Gynecol Obstet.

[8] Bodnar LM, Krohn MA, Simhan HN (2009). Maternal vitamin D deficiency is associated with bacterial vaginosis in the first trimester of pregnancy. J Nutr.

[9] Shimaoka M, Yo Y, Doh K, Kotani Y, Suzuki A, Tsuji I (2019). Association between preterm delivery and bacterial vaginosis with or without treatment. Sci Rep.

[10] Payne SC, Cromer PR, Stanek MK, Palmer AA (2010). Evidence of african-american women’s frustrations with chronic recurrent bacterial vaginosis. J Am Acad Nurse Pract.

[11] Coudray MS, Madhivanan P (2020). Bacterial vaginosis—A brief synopsis of the literature. Eur J Obstet Gynecol Reproductive Biology.

[12] Martens PJ, Gysemans C, Verstuyf A, Mathieu AC. Vitamin D’s Effect on Immune Function. Nutrients. 2020 Apr 28;12(5):1248. doi: 10.3390/nu12051248. 10.3390/nu12051248 PMC7281985 32353972

[13] Bikle DD. Vitamin D regulation of Immune function. Curr Osteoporos Rep. 2022 Jun;20(3):186–93. 10.1007/s11914-022-00732-z. 10.1007/s11914-022-00732-z PMC9065668 35507293

[14] Turner AN, Carr Reese P, Chen PL, Kwok C, Jackson RD, Klebanoff MA (2016). Serum vitamin D status and bacterial vaginosis prevalence and incidence in zimbabwean women. Am J Obstet Gynecol.

[15] Hensel KJ, Randis TM, Gelber SE, Ratner AJ (2011). Pregnancy-specific association of vitamin D deficiency and bacterial vaginosis. Am J Obstet Gynecol.

[16] Allsworth JE, Peipert JF (2007). Prevalence of bacterial vaginosis: 2001–2004 national health and nutrition examination survey data. Obstet Gynecol.

[17] Moore KR, Harmon QE, Baird DD (2018). Serum 25-Hydroxyvitamin D and risk of self-reported bacterial vaginosis in a prospective cohort study of young african american women. J Women’s Health.

[18] Taheri M, Baheiraei A, Foroushani AR, Nikmanesh B, Modarres M (2015). Treatment of vitamin D deficiency is an effective method in the elimination of asymptomatic bacterial vaginosis: a placebo-controlled randomized clinical trial. Indian J Med Res.

[19] Strengthening the reporting. of observational studies in epidemiology for case-control study. Avalable at:https://www.strobe-statement.org/checklists/. [site access: 22 Dec,2022].

[20] World Health Organization: Obesity: Preventing and Managing the Global Epidemic. Report of a WHO Consultation on Obesity. 1998, Geneva: WHO, Geneva, 3–5 June 1997. WHO Technical Report Series 894. 11234459

[21] Sha BE, Chen HY, Wang QJ, Zariffard MR, Cohen MH, Spear GT. Utility of Amsel criteria, Nugent score, and quantitative PCR for Gardnerella vaginalis, Mycoplasma hominis, and Lactobacillus spp. for diagnosis of bacterial vaginosis in human immunodeficiency virus-infected women. J Clin Microbiol. 2005 Sep;43(9):4607–12. 10.1128/JCM.43.9.4607-4612.2005 PMC1234056 16145114

[22] Schlesselman JJ. Case-control studies: design, conduct, analysis. Oxford university press; 1982.

[23] Tabrizi R, Moosazadeh M, Akbari M, Dabbaghmanesh MH, Mohamadkhani M, Asemi Z (2018). High prevalence of vitamin D deficiency among iranian population: a systematic review and meta-analysis. Iran J Med Sci.

[24] Dunlop AL, Taylor RN, Tangpricha V, Fortunato S, Menon R. Maternal vitamin D, folate, and polyunsaturated fatty acid status and bacterial vaginosis during pregnancy. Infectious diseases in obstetrics and gynecology. 2011;2011. 10.1155/2011/216217 PMC3235789 22190843

[25] Klebanoff MA, Turner AN (2014). Bacterial vaginosis and season, a proxy for vitamin D status. Sex Transm Dis.

[26] Ranjit E, Raghubanshi BR, Maskey S, Parajuli P. Prevalence of bacterial vaginosis and its association with risk factors among nonpregnant women: A hospital based study. International journal of microbiology. 2018;2018. 10.1155/2018/8349601 PMC5859802 29692813

[27] Bautista CT, Wurapa E, Sateren WB, Morris S, Hollingsworth B, Sanchez JL (2016). Bacterial vaginosis: a synthesis of the literature on etiology, prevalence, risk factors, and relationship with chlamydia and gonorrhea infections. Military Med Res.

[28] Jespers V, Crucitti T, Menten J, Verhelst R, Mwaura M, Mandaliya K (2014). Prevalence and correlates of bacterial vaginosis in different sub-populations of women in sub-saharan Africa: a cross-sectional study. PLoS ONE.

[29] Guédou FA, Van Damme L, Deese J, Crucitti T, Becker M, Mirembe F (2013). Behavioural and medical predictors of bacterial vaginosis recurrence among female sex workers: longitudinal analysis from a randomized controlled trial. BMC Infect Dis.

